# Scraps

**Published:** 1886-03

**Authors:** 


### SCRAPS

PICKED UP BY THE ASSISTANT-EDITOR.

---

**A Case for Dietetic Reform.**—Guest at a country inn: "I say, landlord, your food is worse than it was last year!" Landlord: "Impossible, sir!"

According to Dr. Brendal *(Peoria Medical Monthly)* "the aim of the practice of medicine may be lucre or bread and butter; but if it be not combined with love of study or the humane desire to do some good, it is the most despicable business imaginable."

**Local Medical Sayings.**—A nurse told a doctor that the medical man who attended her child had said that it died of a tumour on the "remembrances" of the brain. A woman told her clergyman that a doctor had "co-operated" the fact that she had "vertical" veins.

*Life* says, that at last we know why "uneasy lies the head that wears a crown." A newly-arrived chiropodist from the old country announces himself as late corn doctor to the Court of Germany, and tells us he has removed corns from several of the crowned heads of Europe.

*The American Practitioner and News* states that the obstetric fraternity is paralyzed by the announcement that a woman of Valladolid has recently brought forth a litter of seven babies. The mother still lives, with a majority of the infants; but the father is not likely to survive.

"I am just as much opposed to intemperance as anybody," said S.; "but, nevertheless, liquor rightly used is a blessing to humanity. When I was ill last year, I really believe it saved my life." "Very likely," said B.; "but how does that prove that liquor is a blessing to humanity?"

Doctor: "I'm glad to hear, Sandy, that your neighbour who died yesterday was a member of a funeral society." Sandy: "Atweel ay, Doctor. He jist jined it a year sin'. An' there's puir me has been payin' in tae't fur mair than ten o' them without bein' a penny the better o't! He wis aye yin o' the lucky sort sin' he wis born. Puir Jock!"—*Bailie.*

Pliny is authority for the statement that the Romans were 600 years without physic and physicians, and the mortality was very light. The first regular doctor in Rome was Archaghutis, a Greek. He was turned out of the capital city because he was over-fond of using a knife on people, and likewise the actual cautery.—T. C. M., in *Cincinnati Lancet and Clinic.*

The Portuguese authors of the Grammar from which *English as She is Spoke* is culled give this version of the story of the cynical doctor: "A physician, eighty years of age, had enjoyed of a health unalterable. Their friends did him of it compliments every day. 'Mister doctor,' they said to him, 'you are admirable man! What you make them for to bear as well?' 'I will tell you of it, gentlemen,' he was answered them, 'and I exhort you in same time at to follow my example. I live of the product of my ordering without take any remedy who I command to my sicks.'"

**It wasn't the Doctor this time.**—Brown received from his lawyer a few lines on one side of a sheet of note-paper. The writing was so bad that he couldn't make anything out of it. On the way to his office he met

Jones; but their combined efforts were not successful in deciphering it. Jones said, "Come with me;" and entering a chemist's shop, he handed the letter over the counter, saying, "Can you read this?" The chemist looked at it very wisely, retired to the dispensing department, and in a few minutes returned with a bottle of medicine and the letter in a prescription envelope. Here the story ends.

**Bath Baths.**—A 'Society' journal says: "Sir William Jenner has been down at Bath on a brief visit. It is hoped that his appearance heralds the sojourn there of a member of the Royal family. It is certainly a remarkable circumstance that our rheumatic Royalties should put themselves to the trouble and expense of a journey to Aix-les-Bains, when they would find just as comfortable quarters, and at least as efficacious waters, within eighty miles of Windsor. I am sure that the new baths at Bath are far more comfortable and luxurious than anything that can be found abroad; and during the last few months the specialities of the Aix-les-Bains treatment have been successfully introduced there."

Linacre was at the sole expense of founding the Royal College of Physicians of London, for the Crown merely granted the letters-patent. These were issued in 1518, incorporating all physicians in London as one faculty and college, with power to elect a president, to use a common seal, and to hold lands not exceeding the annual value of £12. They were to hold assemblies and govern their faculty in London and within seven miles, all persons being interdicted from practice who did not hold their license. Four censors were to be chosen yearly, for the correction and government of physic and its professors, the examination of medicines, and the punishment of offenders; and physicians were to be exempt from attendance at assizes, inquests, and juries. The power of correction by fine or imprisonment occasioned some embarrassment at a subsequent period, for when some offenders were committed by the College, the gaolers would not receive them into prison, considering the College must charge itself with the custody of its own culprits. To obviate this difficulty, a statute (1 Mary, sess. 2, c. 9) was passed, requiring gaolers to receive persons committed by the College, and also enjoining all justices, mayors, &c., in London to assist the President of the College in searching for faulty apothecary wares.—Bettany's *Eminent Doctors*.

*The Medical Record* (New York) says the beasts of the forest of Ouden, who were learned and skilful in relieving their fellow-creatures' pains, determined to have a General Congress for mutual talk. It gives an account of the President's address, and from its report of the work done some extracts are here made. In the Section on Neurology, the Rabbit related the outcome of his special studies on the "Tendon Reflex in the Hind Extremities of Irritable Mules," and showed several flattened skulls to illustrate the effects and beauty of this phenomenon. In the Section on Diseases of Women and Obstetrics, the Goat opened a debate upon the question, "Whether the Physician should support the Perineum, or the Perineum should support the Physician," which was well maintained. Professor Gulielmus Capricornus read a paper contending that pessaries were a great national blessing, and that all animals should wear them. Professor Suis Ferus reported two thousand operations for sewing up the cervix uteri, with fifteen successful results, upon which he was warmly congratulated. In the Section on Surgery, the Ring-tailed Ape described his new operation for the treatment of intestinal wounds. This consisted in opening the abdomen, removing the whole abdominal contents, and substituting carbolized cotton, thus making the individual thoroughly aseptic.

